# The effect of the video assistant referee (VAR) on referees' decisions at FIFA Women's World Cups

**DOI:** 10.3389/fpsyg.2022.984367

**Published:** 2022-08-12

**Authors:** Yeqin Zhang, Danyang Li, Miguel-Ángel Gómez-Ruano, Daniel Memmert, Chunman Li, Ming Fu

**Affiliations:** ^1^China Football College, Beijing Sport University, Beijing, China; ^2^School of Psychology, Beijing Sport University, Beijing, China; ^3^Faculty of Physical Activity and Sport Sciences (INEF), Polytechnic University of Madrid, Madrid, Spain; ^4^Institute of Exercise Training and Sport Informatics, German Sport University Cologne, Cologne, Germany; ^5^Institute of Physical Education and Training, Capital University of Physical Education and Sports, Beijing, China

**Keywords:** Women's World Cup, video technology, refereeing decision, playing duration, sports performance

## Abstract

Video assistant referee (VAR) has been implemented in women's football, aiming to improve referees' decision-making, but its impact has not yet been analyzed. This study intended to explore how the VAR affects refereeing decisions at Fédération Internationale de Football Association (FIFA) Women's World Cup competitions. The sample includes all 52 matches played in the 2015 tournament before VAR was introduced and all 52 matches played in the 2019 competition where VAR was deployed. For each match, data on ten variables were collected: first half playing time, second half playing time, total playing time, penalties, offsides, fouls, goals, corner kicks, yellow cards, and red cards. The match variables were compared before and after VAR implementation using a Mann–Whitney U test, a Bayesian analysis, a generalized linear model, and a non-clinical magnitude-based inference. The results demonstrated that after VAR was introduced, playing time during the first half [*p* < 0.001, *BF*_10_ = 547.05, Cohen's *d* = 1.06, 90%CI (0.71, 1.40)], the second half [*p* < 0.001, *BF*_10_ = 57.09, Cohen's *d* = 0.91, 90%CI (0.57, 1.25)], and the entire match [*p* < 0.001, *BF*_10_ = 1,120.39, Cohen's *d* = 1.33, 90%CI (0.97, 1.69)] increased significantly with moderate to large effect sizes, while the number of penalties, offsides, and fouls did not vary significantly neither did the number of goals, corner kicks, yellow cards, and red cards. This study has practical implications for professionals in terms of a better understanding of VAR's impact on elite women's football.

## Introduction

The contribution of refereeing decisions to football (soccer) is undeniably important: referees enforce the match's laws and penalize transgressions. On average, referees make 137 observable decisions per match at the international level (Helsen and Bultynck, 2004; Märtins et al., 2022). Any mistake by a referee could impact the match outcome, which is why referees are expected to make the most accurate decisions. However, referees' decision-making process is complex as it entails making instant judgments in fast-paced circumstances involving several players and with limited vision (Lex et al., 2015). In addition, several other factors may also affect referees' decisions, such as perceptual limitations (Oudejans et al., 2000), crowd noise (Unkelbach and Memmert, 2010), fan pressure (Buraimo et al., 2010), match location (Goumas, 2014; Wunderlich et al., 2021), and differences between teams' levels of play (Lago-Peñas and Gómez-López, 2016). Consequently, judgment mistakes and bias in referees' decisions may sometimes seem inevitable.

To fix mistakes and eliminate bias in refereeing decisions, various technical officiating aids have been gradually incorporated into competitive arenas (Collins and Evans, 2012; Kolbinger and Link, 2016; Kolbinger and Lames, 2017). These technologies can be divided into three categories (Kolbinger and Link, 2016): (1) those that enhance perception and assist refereeing decisions (e.g., goal-line technology to identify whether the ball goes over the goal line, Nlandu, 2012); (2) those that substitute human officials for some refereeing decisions (e.g., Hawk-Eye system to determine the ball's bounce point and trajectory, Bal and Dureja, 2012); and (3) those that assist referees in enforcing sports regulations (e.g., vanishing spray to mark the required distance of defending players in football, Kolbinger and Link, 2016). The video assistant referee (VAR), on which this study focuses, is in the first category.

In 2018 the Laws of the Game were amended to include VAR, a match official with independent access to video footage (FIFA, 2019). According to its governing principles, VAR should be used to assist the main referee in reviewing four types of match-changing events where there are “clear and obvious errors” or “serious missed incidents”: goal/no goal, penalty/no penalty, direct red card, and mistaken identity. Generally, VAR automatically checks events in any of these four categories using TV camera footage. If the check demonstrates a possible “clear and obvious error” or “serious missed incident” made by the referees, VAR informs the main referee *via* a headset of what the video depicts and recommends that the main referee review or change the original decision. Normally, there are two kinds of reviews: one is called a VAR-only review, which allows the initial decision to be modified based on the information from the VAR, such as an offside incident that occurred prior to a goal; and the other option is referred to as on-field review, which means the main referee can watch the video footage directly on a monitor next to the field of play before making a final decision (Holder et al., 2021; Spitz et al., 2021).

The use of VAR is helpful in reducing critical errors and improving refereeing accuracy in complex and dynamic football match situations. For example, FIFA (2018) revealed that in the 2018 Men's World Cup, the accuracy rate of refereeing decisions was 95.60% when VAR was not employed and 99.35% when VAR was used. Additionally, in a study of 13 men's national leagues, using VAR was found to increase refereeing decision accuracy from 92.1 to 98.3% (Spitz et al., 2021).

In addition to refereeing accuracy, some studies have investigated the effect of VAR on match variables in men's national and international football competitions. Specifically, compared to other match variables (e.g., goals, corner kicks, yellow cards, and red cards), four variables appear to have been affected by the implementation of VAR:

- Match playing time. Whether a football team can succeed depends on how long it can maintain highly competitive performance throughout the entire match (Maneiro et al., 2020). Related research has observed a significant rise in the match playing time since VAR was introduced (Lago-Peñas et al., 2019, 2020; Han et al., 2020; Kubayi et al., 2022). The main cause of increased playing time is the pauses in the match when VAR intervenes. Each time a review is conducted, the match has to be halted. One study revealed median times of 15.0 s for a VAR-only review and 62.0 s for an on-field review (Spitz et al., 2021). Therefore, matches with VAR intervention generally last longer than do matches without VAR.
- Number of penalty kicks. Given the relatively small number of goals scored in many matches, penalty kicks play an important role in a football match (Makaruk et al., 2020). The number of penalties awarded at the 2018 FIFA Men's World Cup, where VAR was used, was higher than at the 2014 Men's World Cup without VAR (Kubayi et al., 2022). This difference is probably explained by VAR's slow-motion playback function, which could detect fouls that might be missed in fast-paced matches.
- Number of offside judgments. Offside decisions are among the most important responsibilities of a referee, with the potential to dramatically change the outcome of a football match (Helsen et al., 2006). Much of the literature has demonstrated a reduced number of offsides following the introduction of VAR (Lago-Peñas et al., 2019, 2020; Han et al., 2020; Kubayi et al., 2022). One explanation is that VAR corrects erroneous offside calls caused by the flash-lag effect, which refers to the human eye's tendency to detect a moving object as spatially prior to its real position (Baldo et al., 2002; Nijhawan, 2002; Helsen et al., 2006).
- Aggregate of fouls. During a football match, over 30% of the referee's observable decisions involve foul play situations (Spitz et al., 2017). Since the implementation of VAR, the number of fouls in football matches has decreased (Lago-Peñas et al., 2019; Han et al., 2020). This could be explained by players being more cautious in their actions, with a minimum of 12 cameras monitoring the whole pitch. Since any minor physical contact, whether deliberate or unintentional, could be clearly captured on video, players' misconduct may be restrained (Han et al., 2020).

While several previous studies indicated that VAR has a remarkable influence on match variables in men's domestic and international football tournaments (e.g., Lago-Peñas et al., 2019; Han et al., 2020; Spitz et al., 2021; Kubayi et al., 2022), there is still limited scientific research on how VAR may affect women's football competitions. In particular, the effect of VAR at the FIFA Women's World Cup, the most significant tournament for women footballers (Geertsema et al., 2021), has not yet been investigated. Despite the fact that women's football has recently experienced a significant rise in popularity (UEFA., 2017), professionalization (Welford, 2015), and receiving attention from sports researchers and practitioners worldwide (Pfister, 2015), two literature reviews about women's football research (Valenti et al., 2018; Okholm Kryger et al., 2021) clearly indicate that women's football and relevant research is still in development relative to their male counterpart. Therefore, any research regarding women's football will be valuable for broadening the understanding of sport and gender and breaking through the gender constraints (Williams and Hess, 2016). In addition, research on women's football is essential and helpful in accelerating the growth of women's football, which is one of the eleven goals of the FIFA vision 2020–2023 Making Football Truly Global (FIFA., 2020). Beyond that, there is one year to go until the 2023 FIFA Women's World Cup, and a more detailed understanding of VAR's influence on FIFA Women's World Cup tournaments can help the professionals (e.g., referees, coaches, players, and managers) to achieve a higher-quality tournament preparation. Thus, this study took a closer look at the response of women's football toward the implementation of VAR by comparing referee-related variables in the 2015 FIFA Women's World Cup without VAR against those in the 2019 tournament with VAR.

In addition, based on previous research on VAR in men's football, the current study aimed to investigate whether there are similar changes in women's football after VAR implementation. Therefore, two kinds of hypotheses were proposed: first, we assume a significant increase in the playing time and the number of penalties while a significant decrease in the number of offsides and fouls after VAR implementation in the FIFA Women's World Cup, just as it occurs in men's football matches; and second, we assume no significant difference in the number of goals, corner kicks, yellow cards and red cards before and after VAR implementation in FIFA Women's World Cups.

## Materials and methods

### Sample

In the 2015 FIFA Women's World Cup, the total number of matches played was 52, including 36 matches in the group stage and 16 matches in the knockout stage. In the 2019 FIFA Women's World Cup, the match system arrangements were the same, totaling 52 matches consisting of 36 matches in the group stage and 16 matches in the knockout stage. Therefore, all 104 matches from 2015 (no VAR, *n* = 52) and 2019 (VAR, *n* = 52) FIFA Women's World Cup competitions were included in the analysis. The referee-related statistics were compared between the two tournaments.

### Procedures

Consistent with previous research (Kubayi et al., 2022), analysis was undertaken on 10 match variables, all inextricably tied to referees' decisions: first half playing time, second half playing time, total playing time, penalties, offsides, fouls, goals, corner kicks, yellow cards, and red cards. Data for the match variables were gathered from the website of FBref (https://fbref.com), which releases publicly available data through a partnership with the software company StatsBomb. StatsBomb is one of the most recognized companies in the football market (PRNewswire, 2019), supplying statistics and analysis for every European football league and international football tournament (StatsBomb, 2022). To verify the reliability of the data set, the first author independently coded three randomly selected football matches using LongoMatch (version 0.20.8, Barcelona, Spain: https://longomatch.com/en/), a custom-notational analysis system. In the present study, Intraclass Correlation Coefficients (ICCs) between the data provided by StatsBomb and the data provided by the leading author coding were the inter-rater reliability (Koo and Li, 2016). ICC values for 10 match variables ranged from 0.938 to 1, representing excellent reliability (Koo and Li, 2016) (for more details, please see Supplementary Material). The current study was approved by the ethics committee of the local university (BSUCFCIRB-10043070).

### Statistical analyses

The Kolmogorov–Smirnov test revealed that all the match variables were not normally distributed. Hence, the non-parametric Mann–Whitney U test was run to compare differences between each variable with and without VAR. The threshold for statistical significance was set at *p* < 0.05.

As the limitation of traditional frequentist statistics is dependent on *p* values (Vandekerckhove et al., 2018; Wagenmakers et al., 2018), the Bayesian statistical paradigm has been proposed as an alternative method of analysis to reduce the dependence on *p* values (Marsman and Wagenmakers, 2016; Bernards et al., 2017). In this study, a Bayesian Mann–Whitney U test was performed to quantify the relative degree of evidence supporting *H*_0_ (no significant differences before and after VAR implementation) or *H*_1_ (significant differences before and after VAR implementation) through the Bayes factor—*BF*_10_. The subscripts “10” in *BF*_10_ suggest that the model related to *H*_1_ is in the numerator and that the model corresponding to *H*_0_ is in the denominator (Marsman and Wagenmakers, 2016). *H*_0_ would be supported if *BF*_10_ ≤ 0.33, whereas *H*_1_ would be supported if *BF*_10_ ≥ 3.0 (Lee and Wagenmakers, 2013).

For each match variable, a generalized linear model was fitted. The goodness of fit was assessed using the Bayesian information criterion (BIC) and a 95% confidence interval (CI).

Furthermore, the true effects of VAR application on each match variable were analyzed using non-clinical magnitude-based inference (Hopkins et al., 2009). Differences were determined by computing standardized effect sizes, reported as Cohen's *d* with 90%CI. The thresholds of effect size for small, moderate, large, very large, and extremely large were 0.2, 0.6, 1.2, 2.0, and 4.0 (Hopkins et al., 2009). The smallest worthwhile change was estimated as 0.2 with standardized units. Unless the 90%CI encompassed both positive and negative values, an effect was regarded as clear. The qualitative likelihood of clear effects was measured on the following scale: <0.5% for *most unlikely*, 0.5–5% for *very unlikely*, 5–25% for *unlikely*, 25–75% for *possibly*, 75–95% for *likely*, 95–99.5% for *very likely*, and > 99.5% for *most likely* (Hopkins et al., 2009).

IBM SPSS statistical software (version 26.0), JASP software (version 0.16.1), and Microsoft Excel (Hopkins, 2007) were used for all statistical analyses.

## Results

Descriptive data of the match variables in FIFA Women's World Cup tournaments are summarized in Table 1. Following the implementation of VAR, a significant rise was observed in playing time in the first half (*p* < 0.001, *BF*_10_ = 547.05), the second half (*p* < 0.001, *BF*_10_ = 57.09), and the full match (*p* < 0.001, *BF*_10_ = 1120.39). While the other match indicators did not show a significant change after the intervention of VAR.

**Table 1:** Descriptive statistics and results of Mann–Whitney U test and Bayesian analysis for match variables without VAR (2015) and with VAR (2019).

**Variables**	**No VAR (2015)**		**VAR (2019)**		***Z***	***p*-value**	***BF*_10_**	**Cohen's *d* with 90%CI**
	***M***	***SD***	***M***	***SD***				
First half playing time	46.12	1.10	47.37	1.25	−5.134	<0.001	547.05	1.06 (0.71, 1.40)
Second half playing time	48.42	0.78	49.46	1.41	−4.360	<0.001	57.09	0.91 (0.57, 1.25)
Total playing time	94.54	1.41	96.83	1.98	−5.796	<0.001	1120.39	1.33 (0.97, 1.69)
Penalties	0.42	0.72	0.50	0.61	−1.119	0.263	0.27	0.11 (−0.21, 0.44)
Offsides	3.50	2.02	3.90	2.39	−0.810	0.418	0.27	0.18 (−0.14, 0.51)
Fouls	22.85	7.40	22.17	6.77	−0.430	0.667	0.22	−0.09 (−0.42, 0.23)
Goals	2.69	2.02	2.65	1.98	−0.140	0.889	0.23	−0.02 (−0.34, 0.30)
Corner kicks	9.67	3.94	9.13	3.58	−0.529	0.596	0.26	−0.14 (−0.47, 0.18)
Yellow cards	2.13	1.47	2.44	1.38	−1.347	0.178	0.33	0.22 (−0.11, 0.54)
Red cards	0.06	0.24	0.08	0.27	−0.389	0.697	0.27	0.08 (−0.25, 0.40)

*M, the mean value of the match variable; SD, the standardized deviation of the match variable; BF, the Bayes factor; CI, the confidence interval.*

The results of the generalized linear model for each match variable are shown in Table 2. VAR implementation resulted in a statistically significant increase in playing time in the first half (*p* < 0.001), the second half (*p* < 0.001), and consequently, the entire match (*p* < 0.001). In contrast, there was no significant change in the other match variables after the intervention of VAR.

**Table 2:** Results of the generalized linear model for each match variable.

**Variables**	**Estimate**	**95%CI**	***p*-value**	**BIC**
First half playing time	1.250	(0.802, 1.698)	<0.001	340.996
Second half playing time	1.038	(0.606, 1.471)	<0.001	333.564
Total playing time	2.288	(1.635, 2.942)	<0.001	419.376
Penalties	0.077	(−0.178, 0.332)	0.554	223.536
Offsides	0.404	(−0.439, 1.246)	0.347	472.275
Fouls	−0.673	(−3.373, 2.027)	0.625	714.518
Goals	−0.038	(−0.799, 0.722)	0.921	450.933
Corner kicks	−0.538	(−1.970, 0.894)	0.461	582.638
Yellow cards	0.308	(−0.234, 0.850)	0.266	380.601
Red cards	0.019	(−0.077, 0.115)	0.695	21.030

*CI, the confidence interval; BIC, the Bayesian information criterion.*

**Figure 1:**
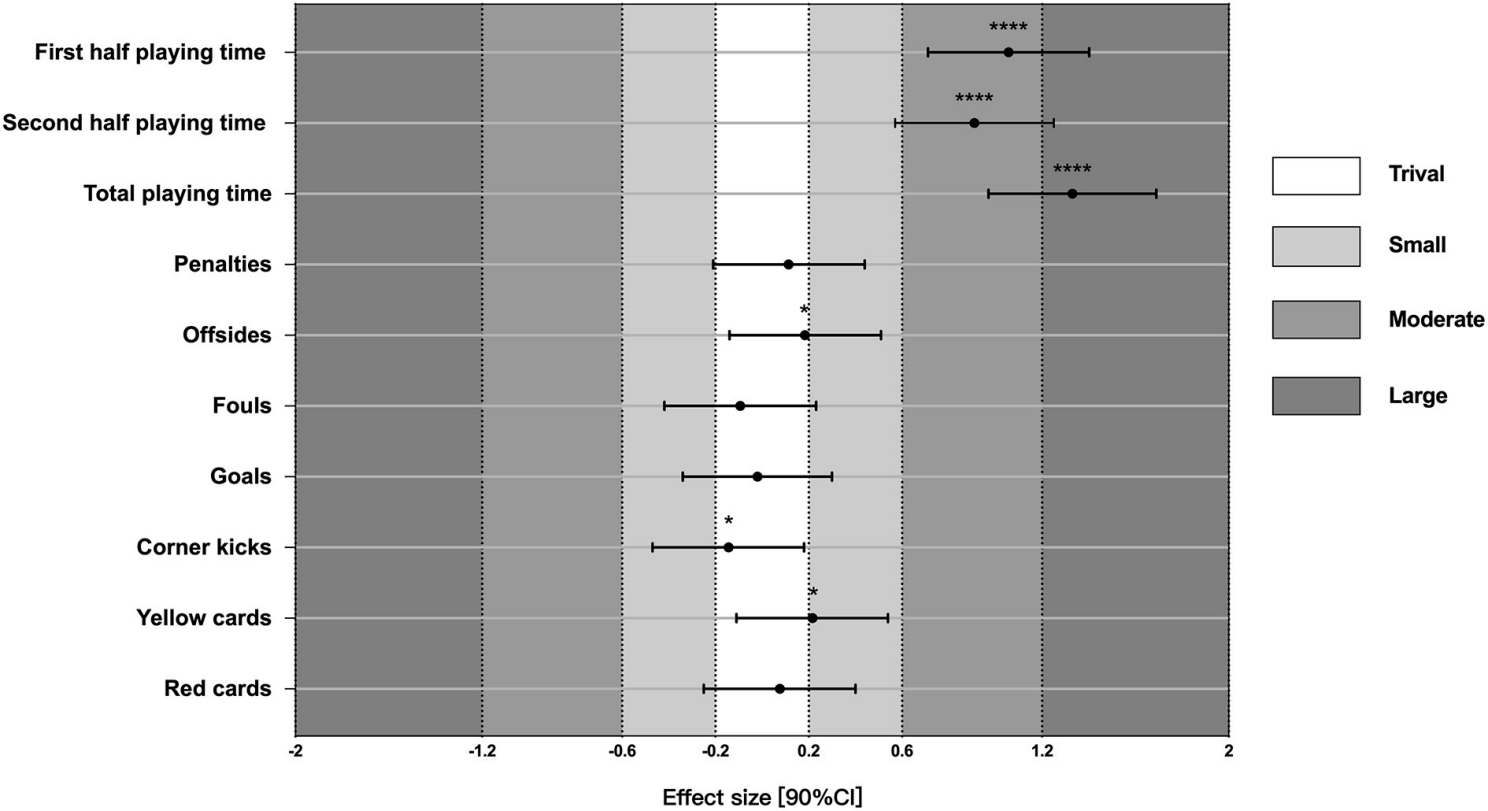
Effect sizes of differences in each match variable before and after the introduction of VAR. Asterisks indicate the qualitative likelihood of the magnitude of true effects: ^*^possibly; ^****^most likely; CI, confidence interval.

The true effects of VAR application on each match variable, as determined by non-clinical magnitude-based inference (Hopkins et al., 2009), are illustrated in Figure 1. The findings reveal that before and after VAR was introduced, only three variables showed clear effects: playing time in the first half [*d* = 1.06, 90%CI (0.71, 1.40)], the second half [*d* = 0.91, 90%CI (0.57, 1.25)], and the entire match [*d* = 1.33, 90%CI (0.97, 1.69)], with *most likely* moderate to large effect sizes. Conversely, the rest of these variables showed an unclear effect as their 90%CIs of Cohen's *d* results included both positive and negative values.

## Discussion

VAR has been introduced progressively into football. As the usage of VAR in women's football has not previously been assessed, this study adds new information to the evidence base on VAR's effects in elite football, particularly in women's football. The main finding is that the playing duration in each match was significantly longer, on average, at the 2019 FIFA Women's World Cup with VAR than at the 2015 tournament without VAR.

Corresponding to our first hypothesis, the amount of time spent on the field increased significantly in both halves and, consequently, for the entire match, following VAR's introduction. These findings are consistent with previous findings for men's football (Lago-Peñas et al., 2019, 2020; Han et al., 2020; Kubayi et al., 2022). Some critics of VAR in football have argued that by halting play to review footage of disputed incidents, the match's flow is broken, and its tempo slowed (Nlandu, 2012; Svantesson, 2014; Scanlon et al., 2022). However, this study finds that the time added to a match by using VAR is not very long in the FIFA Women's World Cup: only around one minute in the first half (46.12 min without VAR vs. 47.37 min with VAR) and the second half (48.42 vs. 49.46 min), respectively, and consequently two minutes in the full match (94.54 vs. 96.83 min). These findings imply that implementing VAR does not dramatically lengthen the duration of a football match, providing support for previous studies (Errekagorri et al., 2020; Lago-Peñas et al., 2020).

Contrary to our first hypothesis, the number of offsides did not significantly change following the implementation of VAR. This result is inconsistent with the considerable decrease in the number of offsides found in men's football after VAR's introduction, both in the FIFA Men's World Cup (Kubayi et al., 2022) and domestic leagues (Lago-Peñas et al., 2019; Han et al., 2020). There are two possible explanations for the inconsistency. The first concerns the flash-lag effect (Nijhawan, 2002), which is often cited to explain errors in offside calls (Helsen et al., 2006) that VAR can effectively correct. Motion speed influences the flash-lag effect, which tends to be smaller at slower speeds (Shioiri et al., 2010). Generally, women football players have a slower sprint speed than men football players (de Araújo et al., 2020; Haugen et al., 2020), meaning that the flash-lag effect may be less significant in women's football matches than in men's matches. Consequently, after introducing VAR, the reduction in the number of offside calls in women's matches is not as significant as that in men's matches. Second, compared to men's football matches, women's football matches create less favorable patterns of match structure and tend to have fewer penetrative passes (Tenga et al., 2017). As penetrative passes are associated with both scoring opportunities and offside possibilities, fewer penetrative passes in women's football leave less scope for intervention by VAR in correcting offside calls by the assistant referees. These differences may reflect that women's football matches have their own styles of play and idiosyncrasies compared to men's football (Gutierrez and García-López, 2012; Bradley et al., 2014).

Inconsistent with our first hypothesis, this study found that the number of fouls and penalties did not significantly change after VAR was implemented. These results are contrary to previous findings in men's football, where VAR use has resulted in referees awarding fewer free kicks for fouls (Lago-Peñas et al., 2019; Han et al., 2020) and more penalties (Kubayi et al., 2022). The inconsistencies could be attributable to women football players being less aggressive than men football players (Coulomb-Cabagno et al., 2005; Coulomb-Cabagno and Rascle, 2006), manifesting in fewer rule violations, such as illegal tackles, interceptions, and striking of opponents. Consequently, VAR plays a relatively small part in addressing misconduct in women's football matches.

Corresponding to our second hypothesis, a non-significant change in the number of goals, corner kicks, and yellow/red cards was found following the implementation of VAR, which was consistent with previous studies related to men's football (Han et al., 2020; Lago-Peñas et al., 2020). A likely explanation for this is that these variables are generally less affected by referees' visual limitations compared to the judgment of offside; thus, the misjudgment of these variables is relatively rare. Therefore, these indicators did not show considerable change before and after the intervention of VAR in FIFA Women's World Cup tournaments.

There are certain limitations in this study that need to be addressed in future research. First, between the 2015 FIFA Women's World Cup and the 2019 tournament, there were developments in techniques and tactics of modern football that may have influenced the studied match variables. Although this study focused on referee-related indicators that are relatively less sensitive to such developments, future studies of the impact of VAR should also consider how techniques and tactics have evolved. Second, according to Lago-Peñas and Gómez-López (2016), the greater the difference in score between the two teams, the less stoppage time the referee adds to the second half of the match; in close matches, by contrast, added time tends to be longer when a higher-ranking team is trailing than when it is leading. Consequently, the strength differences between opposing teams should also be considered in future research. Lastly, as this study only considered a relatively small number of women's football matches, caution must be exercised in interpreting our findings on the effects of VAR implementation on referees' decisions. Future research should analyze a greater number of women's domestic and international football competitions to test the robustness of this study's findings and increase the statistical power.

Regarding practical applications, this study highlights the importance of examining novel technical refereeing devices in women's international football. The findings may aid football practitioners (e.g., referees, coaches, players, and managers) in amply comprehending how VAR has affected elite women's football and in effectively identifying tactics for improving team performance. VAR does not appear to dramatically affect elite women's football in terms of match duration. Nonetheless, VAR should be improved to minimize disruptions to the matches' flow and rhythm. Furthermore, with the development of modern football, demands on women football players are increasing, for instance, through greater emphasis on high-speed running and sprinting (Griffin et al., 2020), passing accuracy (Soroka and Bergier, 2010), and high levels of anaerobic qualities and aerobic capacity (Turner et al., 2013). These new challenges will reinforce the role of VAR in women's football matches.

## Conclusion

This study investigated the influence of VAR on referee-related match variables at FIFA Women's World Cup tournaments. The primary findings were that playing time in the first half, second half, and full match increased significantly but not excessively. These findings indicate that the introduction of VAR has not excessively impacted elite women's football matches. Nonetheless, it is necessary to continue analyzing the effects of this innovative refereeing aid on women's football to verify its effectiveness and assess its applicability.

## Data availability statement

Publicly available datasets were analyzed in this study. This data can be found here: https://fbref.com.

## Author contributions

YZ and DL contributed to the conception and design of the study, performed the statistical analysis, and wrote the first draft of the manuscript. YZ organized the database. M-ÁG-R, DM, CL, and MF reviewed and revised the manuscript. All authors have made a substantial and direct contribution to the manuscript and approved its final version.

## Funding

This work was supported by the Fundamental Research Funds for the Central Universities (Grant No. 20211022), the China Scholarship Council (Grant No. 202106520014), and the Sport Sciences Network (2022: 25/UPB/22 SPAA, Sports Performance Analysis Association).

## Conflict of interest

The authors declare that the research was conducted in the absence of any commercial or financial relationships that could be construed as a potential conflict of interest.

## Publisher's note

All claims expressed in this article are solely those of the authors and do not necessarily represent those of their affiliated organizations, or those of the publisher, the editors and the reviewers. Any product that may be evaluated in this article, or claim that may be made by its manufacturer, is not guaranteed or endorsed by the publisher.
