# Supplementary material for: The effect of the video assistant referee (VAR) on referees' decisions at FIFA Women's World Cups
Source: Front Psychol. 2022 Aug 12;13:984367. doi: 10.3389/fpsyg.2022.984367 (PMC9413155; doi:10.3389/fpsyg.2022.984367)
Supplement: Supplementary file 1 [file Data_Sheet_1.docx]

Supplementary Material

# How to Verify the Reliability of the Date Set?

First, all 104 matches from 2015 (*n* = 52) and 2019 (*n* = 52) FIFA Women’s World Cup competitions were numbered from 1 to 104. Then we used the Random Number Generator (https://www.calculator.net/random-number-generator.html) to create three random integers, after setting the lower limit (1) and the upper limit (104). In the present study, the Random Number Generator generated three random numbers: 79, 93, and 95. According to the three numbers, matches corresponding to the numbers were selected (for more information on the three matches, please see Table S1).

Second, following the practice of Stone et al. (2021), the first author independently coded the above three randomly selected football matches using the LongoMatch (version 0.20.8, Barcelona, Spain. Available online: https://longomatch.com/en/), a custom-notational analysis system, to examine ten indicators which were playing time during the first half, playing time during the second half, total playing time, penalties, offsides, fouls, goals, corner kicks, yellow cards, and red cards. The ten indicators are selected based on previous research (Kubayi et al., 2022).

Third, ICC indicates the Intraclass Correlation Coefficient, a widely used reliability index in test-retest, intra-rater, and inter-rater reliability analyses (Koo and Li, 2016), and the values of ICC less than 0.5, between 0.5 and 0.75, between 0.75 and 0.9, and greater than 0.90 are indicative of poor, moderate, good, and excellent reliability, respectively (Koo and Li, 2016). In the present study, the ICC between the data provided by StatsBomb and the data provided by the leading author coding is the inter-rater reliability, which reflects the variation across raters that evaluate the same group of subjects (Stolarova et al., 2014). According to Table S2, we could find that the ICC ranges from 0.938 to 1.000 on the ten indicators, representing excellent reliability (Koo and Li, 2016).

**Table S1.** The information of three randomly selected football matches.

| No. | Match teams | Match date | Match stage |
| --- | --- | --- | --- |
| 79 | Nigeria vs. France | 17 June 2019, 21:00 (venue time) | Group stage |
| 93 | Spain vs. the USA | 24 June 2019, 18:00 (venue time) | Knockout stage |
| 95 | Italy vs. China | 25 June 2019, 18:00 (venue time) | Knockout stage |

**Table S2.** The ICCs between StatsBomb and Author on ten match indicators.

| Indicators | No.79 Nigeria vs. France | | No.93 Spain vs. the USA | | No.95 Italy vs. China | | ICC |
| --- | --- | --- | --- | --- | --- | --- | --- |
|  | StatsBomb | Author | StatsBomb | Author | StatsBomb | Author |  |
| Playing time first half | 47 | 47 | 48 | 48 | 48 | 48 | 1.000 |
| Playing time second half | 53 | 53 | 52 | 52 | 49 | 49 | 1.000 |
| Total playing time | 100 | 100 | 100 | 100 | 97 | 97 | 1.000 |
| Penalties | 1 | 1 | 2 | 2 | 0 | 0 | 1.000 |
| Offsides | 1 | 1 | 4 | 3 | 4 | 4 | 0.938 |
| Fouls | 25 | 24 | 25 | 24 | 19 | 18 | 0.960 |
| Goals | 1 | 1 | 3 | 3 | 2 | 2 | 1.000 |
| Corner kicks | 11 | 11 | 5 | 5 | 9 | 9 | 1.000 |
| Yellow cards | 4 | 4 | 2 | 2 | 0 | 0 | 1.000 |
| Red cards | 1 | 1 | 0 | 0 | 0 | 0 | 1.000 |

*Note*. StatsBomb indicates the data provided by the StatsBomb company, which can be found at: <https://fbref.com>; Author indicates the data provided by the leading author, who independently coded the three randomly selected football matches using the LongoMatch notational analysis system; ICC indicates the inter-rater reliability between the data provided by StatsBomb and the data provided by the leading author coding.

**References**

Koo, T.K., and Li, M.Y. (2016). A guideline of selecting and reporting intraclass correlation coefficients for reliability research. *J. Chiropr. Med.* 15(2)**,** 155–163. doi: 10.1016/j.jcm.2016.02.012

Kubayi, A., Larkin, P., and Toriola, A. (2022). The impact of video assistant referee (VAR) on match performance variables at men’s FIFA World Cup tournaments. *Proc. Inst. Mech. Eng. P: J. Sports Eng. Technol.* 236(3)**,** 187–191. doi: 10.1177/1754337121997581

Stolarova, M., Wolf, C., Rinker, T., and Brielmann, A. (2014). How to assess and compare inter-rater reliability, agreement and correlation of ratings: an exemplary analysis of mother-father and parent-teacher expressive vocabulary rating pairs. *Front. Psychol.* 5**,** 509. doi: 10.3389/fpsyg.2014.00509

Stone, J.A., Smith, A., and Barry, A. (2021). The undervalued set piece: analysis of soccer throw-ins during the English Premier League 2018–2019 season. *Int. J. Sports Sci.* *Coach*. 16(3)**,** 830–839. doi: 10.1177/1747954121991447
